# Substrate-to-inoculum ratio drives solid-state anaerobic digestion of unamended grape marc and cheese whey

**DOI:** 10.1371/journal.pone.0262940

**Published:** 2022-01-27

**Authors:** Josue Kassongo, Esmaeil Shahsavari, Andrew S. Ball

**Affiliations:** ARC Training Centre for the Transformation of Australia’s Biosolids Resource, School of Science, RMIT University, Melbourne, VIC, Australia; Tsinghua University, CHINA

## Abstract

Inoculation dose is a key operational parameter for the solid-state anaerobic digestion (SS-AD) of lignocellulosic biomass, maximum methane recovery, and stable digester performance. The novelty of this study was the co-digestion of unamended full-strength grape marc and cheese whey for peak methane extraction at variable inoculation levels. An acclimatised digestate from a preceding anaerobic treatment was used as a downstream inoculum. The impact of inoculum size (wet weight) was evaluated at 0/10, 5/5, 7/3 and 9/1 substrate-to-inoculum (S/I) ratios, corresponding to an initial concentration of 20–30% total solids (TS) in digesters over 58 days at 45°C. The optimal 7/3 S/I produced the highest cumulative methane yield, 6.45 L CH_4_ kg^-1^ VS, coinciding with the lowest initial salinity at 11%; the highest volumetric methane productivity rate of 0.289±0.044 L CH_4_ L_Work_^-1^ d^-1^; the highest average COD/N ratio of 9.88; the highest final pH of 9.13, and a maximum 15.07% elemental carbon removal; for a lag time of 9.4 days. This study identified an optimal inoculation dose and opens up an avenue for the direct co-digestion of grape marc and cheese whey without requirements for substrate pretreatment, thus improving the overall bioenergy profile of the winery and dairy joint resource recovery operations.

## Introduction

Anaerobic digestion (AD) of lignocellulosic biomass is a technology for waste management whilst producing renewable energy from diverse recalcitrant feedstocks which include corn stover [1, 2]; wheat straw [3]; yard trimmings [4]; forestry wastes [5] and energy crops [6]. Generally, the treatment of lignocellulosic material is conducted in solid-state AD (SS-AD) systems for higher volumetric methane productivity and lower water utilisation [7]. Based on the dry weight (total solids, TS) of organic material, AD reactors are categorised as wet (≤ 10% TS), semi-dry (10–20% TS), and dry (≥20% TS) systems [8, 9]. In liquid AD technology, organics are completely submerged, requiring large digestion tanks with additional costs associated with slurry heating and homogenisation [5, 10]. In contrast, dry-based AD significantly reduces water utilisation and digestion reactor sizes. However, organic overloading may lead to fast acidification and reactor failure at high solids concentration [10].

Among essential operational parameters for stable digestion, temperature of treatment, nutritional balance of feedstock and substrate-to-inoculum (S/I) ratio are key factors in SS-AD systems [11, 12]. In addition, the effectiveness of the bioenergy generation is largely governed by the microbial community. Consequently, anaerobic treatments regularly use organic matter with a microbial content, such as animal manure or wastewater treatment sludge; the latter has been the most commonly used inoculum for rapid reactor start-ups [13, 14].

The advantages of thermophilic-digestion include seed deactivation and digestate sanitation before land application [15, 16]. Moreover, thermophilic digestion temperatures have been credited with faster reaction kinetics and increased methane yields; however, rapid hydrolysis results in the accumulation of ammonia and volatile fatty acids, lowering the pH and methane productivity [17, 18]. More recently, Hupfauf et al. [19] concluded that 45°C may optimise the process efficiency of biomethanation and support bio-control of the effluent for downstream agricultural uses, thus combining the benefits of both the mesophilic and thermophilic temperature regimes.

The use of co-substrates in the treatment offers dilution of toxic compounds, improvement of the COD/N ratio and additional microbial synergisms [20–23]. An effective nutritional balance supports the development of resilient bacterial consortia, capable of withstanding physicochemical and operational changes [24]. Globally, the agri-industrial sector generates considerable organic wastes that require sustainable treatment, circular utilisation and ultimately efficient disposal. For example, standard cheese-manufacturing operations process on average 10 L of fresh milk for 1 kg of cheese produced, resulting in 9 L of high-strength liquid effluent (known as cheese whey, CW) [25]. Globally, cheese whey production is estimated at some 200 million tonnes, annually [26]. In addition, the grape industry harvests nearing 80 million tonnes per year, worldwide. Approximately 75% of the grapes harvested are channelled to wine production, resulting in an estimated 20% of solid wastes (known as grape marc, GM) for every unit volume of grape crushed [27].

Overall, the environmental impact of the dairy and wine industries can be substantial where waste valorisation systems are not utilised. Globally, as concluded by Prazeres et al. [25], the implementation and running costs of valorisation technologies are prohibitive for small- to medium-sized industries. However, such wastes need to be considered as a low-cost feedstock for secondary industries geared at resource recovery and bioenergy generation. The co-digestion of grape marc (carbon-rich) and cheese whey (nitrogen-rich) for methane production achieves the objectives of energy production and remediation, providing a compact solution for the two applicable agri-industries whilst limiting the complexity in organics composition that come along when processing diverse wastes [28].

The S/I ratio has been identified amongst critical factors for the establishment of economically viable large-scale digesters [7, 29]. The S/I ratio is linked to the specificity of the waste composition and digestion conditions. The refining of the bacterially-mediated AD by manipulation of the inoculum dose, thus combining the optimal bio-catalytic capability with the required accessible nutrients, is known to reduce the lag time of biogas production and improve the bioenergy profile of waste treatment [30, 31]. The S/I for lignocellulosic materials is routinely reported in the range of 1–6, on a volatile solids basis [1, 12, 30, 32]. However, the S/I is influenced by several parameters including inoculum source, substrate type and digestion conditions [12, 33, 34].

To the best of the authors’ knowledge, determination of optimal S/I for the co-digestion of grape marc and cheese whey has not been conducted previously. Therefore, this study aimed to investigate the optimal S/I ratio for the treatment of grape marc and cheese whey without any pretreatment and to evaluate the physicochemical transformations in the digestate and how these impacted bioenergy production.

## Materials and methods

### Wastes characterisation

Dried spent grape marc (GM) that had undergone prior distillation for alcohol recovery was sourced from Tarac Technologies, Australia. Cheddar cheese whey (CW) was sampled from Saputo Dairy Division, Australia. Feedstocks were stored at 4°C until use to avoid initiating unwanted microbial activity [31]. The inoculum was sampled in a fill-and-draw approach from an active laboratory-scale digester of composition 3/1 grape marc and cheese whey, respectively, operating at 45°C; on day 120.

Characterisation of the parameters of individual feedstock was conducted in triplicate on samples prior to anaerobic digestion (Table 1). Conductivity and salinity were determined with the use of a Compact Conductivity Meter (LAQUAtwin-CC-11, HORIBA Scientific) and Compact Salt Meter (LAQUAtwin-Salt-11, HORIBA Scientific), respectively. A HANNA Instruments edge pH meter was used to measure pH. Total solids, COD and total Kjeldahl nitrogen (TKN) were determined according to Standard Methods [35].

**Table 1:** Characteristics of unmixed agri-industrial feedstock and inoculum before anaerobic digestion.

Feedstock Parameters	Grape^a^ Marc	Cheese^a^ Whey	Sludge^a^ Inoculum
TS (%)	38.7 ±1.51	7.87 ±1.02	21.5 ±0.07
VS (%)	24.1 ±0.54	3.80 ±0.88	15.1 ±1.82
CODt (g L^-1^)	223 ±16.3	67.1 ±3.01	101 ±7.23
CODs (g L^-1^)	48 ±12	48 ±5.7	13 ±0.2
EC (mS cm^-1^)	15 ±0.2	14 ±0.3	15.6 ±0.12
Salinity (%)	5.20 ±0.32	13.9 ±0.11	9.8 ±0.1
pH	9.19 ±0.01	5.41 ±0.01	7.91 ±0.16
TKN (g L^-1^)	51.8 ±0.76	11.5 ±0.16	2.42 ±0.32

^a^ Data recorded as mean ± standard error; TS, total solids; VS, volatile solids; CODt, total COD; CODs, soluble COD; TKN, total Kjeldahl nitrogen; EC, electrical conductivity.

### Elemental characterisation

For elemental analyses (CHNOS), samples of unmixed feedstock and inoculum were incubated at 70°C for 24 h, then ground to a fine powder. Characterisation of the input substrates was performed by the Chemical Analysis Facility, Macquarie University, Australia. Briefly, dried samples were loaded into tin containers of oxidisable metal and heated to 970°C in the presence of helium. Individual elements were determined by frontal gas chromatography using a standard curve of National Institute of Standards and Technology (NIST, USA) primary standards. The instrumentation system included Vario MICRO cube elemental analysers (Elementar Analysensysteme GmbH, Germany), applicable software and a micro balance. The same CHNOS method was applied to samples at varying S/I ratios before AD and with the digestate (after AD). Generally, the elemental composition is given by % mass, and converted to % molar by dividing the mass by the atomic mass of each element. Finally, the results are divided by the % molar of the nitrogen to obtain the biomass chemical formula. Results were further used to determine the general molecular formula and both the maximum theoretical biogas and methane production potentials through the Buswell and Neave [36] stoichiometry equation:

$$C_{a}H_{b}O_{c}N_{d}+\left(a-\frac{b}{4}-\frac{c}{2}+\frac{3d}{4}\right)*H_{2}O\to \left(\frac{4a+b-2c-3d}{8}\right)\;*{CH}_{4\;}+\left(\frac{4a-b+2c+3d}{8}\right)*{CO}_{2}+d*{NH}_{3}$$

The determination of stoichiometric coefficients (a, b, c, and d) assumes that all biodegradation reactions within the digestate go to completion [36, 37]. Therefore, the calculation of the maximum theoretical biogas (B_th_) and methane (M_th_) were calculated using equations [Eq (1)] and [Eq (2)], respectively [37]:

$$B_{th}\left[\frac{m^{3}}{kg\;VS}\right]=\frac{a*22.415}{12a+b+16c+14d}$$ (1)

$$M_{th}\left[\frac{m^{3}}{kg\;VS}\right]=\frac{\frac{\left(4a+b-2c-3d\right)}{8}*22.415}{12a+b+16c+14d}$$ (2)

### Reactor setups

Reactants underwent fermentation in customised glassware reaction bottles of 310 mL total volume, arranged in parallel, sparged with stock nitrogen gas for 2 minutes, and then sealed with a rubber stopper. The biogas produced flowed externally through a 6 mm clear vinyl tubing into water-filled cylinders for mass transfer determination. Duplicate batch reactor setups of constant substrate feedstock mixing ratio at 3/1 GM/CW (w/w) were configured in parallel. For a relatively unknown GM/CW substrate co-digestion or to mitigate possible inhibition, it is recommended to test three to four levels of S/I ratio [11]. Therefore, the feedstock (wet weight) was inoculated at variable S/I ratios of 5/5, 7/3 and 9/1, including blank assays without substrate at 0/10 S/I with an overall working volume of 100 mL. An acclimatised digestate from a previous GM/CW anaerobic treatment was used as downstream inoculum. Incubation was conducted at 45°C over 58 days. The headspace volume within the reactors was 210 mL. Weekly biogas volumetric production was measured using water displacement [38, 39]. Biogas was measured at ambient temperature and sampled for compositional analyses (CH_4_, CO_2_ and O_2_); gas composition was measured daily for the initial two weeks of operation and subsequently twice a week with the use of GEM2000 Landfill Gas Analyser (Geotech, UK). Dry biogas in the normal state was obtained by correcting wet biogas according to standard temperature (0°C) and pressure (101.325 kPa) and expressed as NL gas kg^-1^ VS [12].

### Specific methane production (SMP)

The SMP of each digestion setup corresponded to the cumulative methane fraction of the cumulative biogas expressed as a function of the VS_fed_, as digestion progressed [9]. Replicate setups of the corresponding S/I ratio were averaged and reported as mean ± standard error values. SMP is expressed as L CH_4_ kg^-1^ VS [40, 41], and calculated according to equation [Eq (3)] [12]:

SMP=V1W (3)

where *V1* is the cumulative methane volume (L) during the entire digestion period, and *W* is the weight (kg) of VS substrate added to the digester.

The methane production was also normalised to standard temperature and pressure (STP) conditions and expressed as NL CH_4_ kg^-1^ VS [11].

### Volumetric methane productivity rate (VMPR)

The VMPR is the volume of methane (wet) accumulated in the headspace volume per unit working volume of the reactor at a particular time. Volumetric methane productivity rate represents the workable energy recovered in the cubic volume occupied by the reactants. VMPR is expressed as L CH_4_ L_Work_^-1^ d^-1^ ([12]) and calculated according to equation [Eq (4)]:

VMPR=V1(V2*T80) (4)

where *V2* is the reactor working volume (L_Work_), and *T*_*80*_ is the shortest technical digestion time (d) calculated according to the time for the cumulative methane volume to achieve 80% of *V1*.

### Statistical analyses

Analysis of variance (ANOVA) of repeated biogas measurements and physicochemical duplicates were utilised to determine significance (p < 0.05) of variations. Groups of varying S/I ratio were separately analysed before and after treatment.

### Kinetic simulations

Common kinetic models were used to better assess SMP curves. To describe the methanation process, non-linear regressions were utilised [40, 42, 43], thus the first-order equation [Eq (5)]:

$$B\left(t\right)=B_{0}*\left[1-exp\left(-kt\right)\right]$$ (5)

where *B(t)* is the cumulative methane volume (L CH_4_ kg^-1^ VS) at a digestion time *t* (d); *B*_*0*_ is the methane potential of the substrate material (L CH_4_ kg^-1^ VS); *k* is the first-order disintegration rate constant (d^-1^); *t* is the digestion time (d).

To estimate the lag phase, the modified Gompertz model was simulated [Eq (6)] [43]:

$$B\left(t\right)=B_{0}*exp\left\{-exp\left[\left(R_{m}*\frac{exp}{B_{0}}\right)*\left(\lambda -t\right)+1\right]\right\}$$ (6)

where *R*_*m*_ is the maximal methane production rate (L CH_4_ kg^-1^ VS d^-1^); *λ* is the lag phase (d); all mathematical models were simulated with the Solver tool of Microsoft Office Excel.

## Results and discussion

### Biogas production

**Fig 1:**
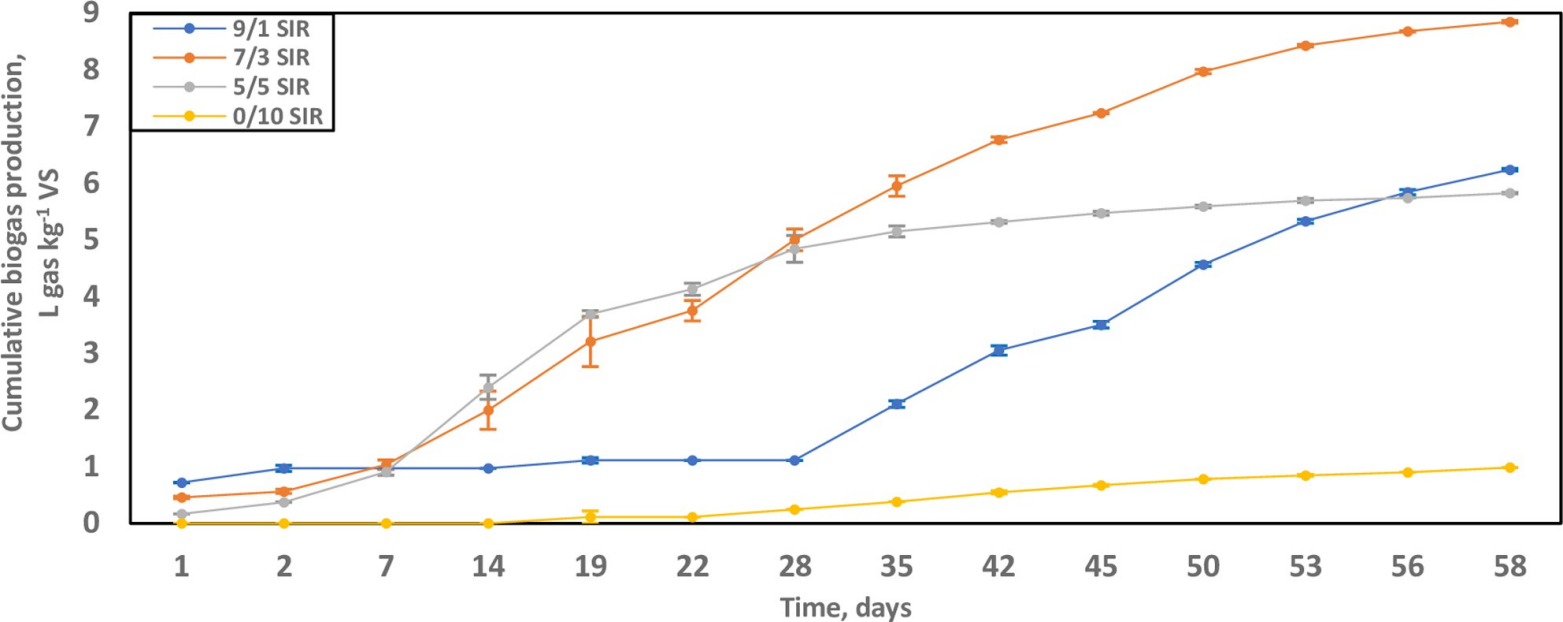
Biogas production curves (L gas kg^-1^ VS) of digestion setups at 45°C over 58 days. The substrate-to-inoculum (S/I) ratios were 0/10 [yellow]; 5/5 [grey]; 7/3 [orange]; and 9/1 [blue]. Data are presented as averages with standard error.

This study pertained to the treatment of unamended raw wastes in anaerobic digesters at 45°C over 58 days. The digestion containing grape marc/cheese whey S/I ratio of 7/3 (wet weight basis) showed the highest cumulative biogas production, reaching 8.85 L gas kg^-1^ VS (8.15 NL gas kg^-1^ VS when normalised to STP) (Fig 1). The maximum cumulative biogas yields over increasing substrate concentration reached a peak value at 7/3 S/I ratio; before lowering on the 9/1 S/I ratio reactors at 6.24 L gas kg^-1^ VS (5.75 NL gas kg^-1^ VS when normalised to STP).

The residual biogas production potential of microbes was at the 0/10 S/I ratio where nutrient was limiting, only reaching 0.98 L gas kg^-1^ VS (0.91 NL gas kg^-1^ VS in STP). In reactors containing 5/5 S/I ratio i.e. equal grape marc and cheese whey, the cumulative biogas accrued to 5.83 L gas kg^-1^ VS (5.37 NL gas kg^-1^ VS in STP) (Fig 1).

### Methane production

**Fig 2:**
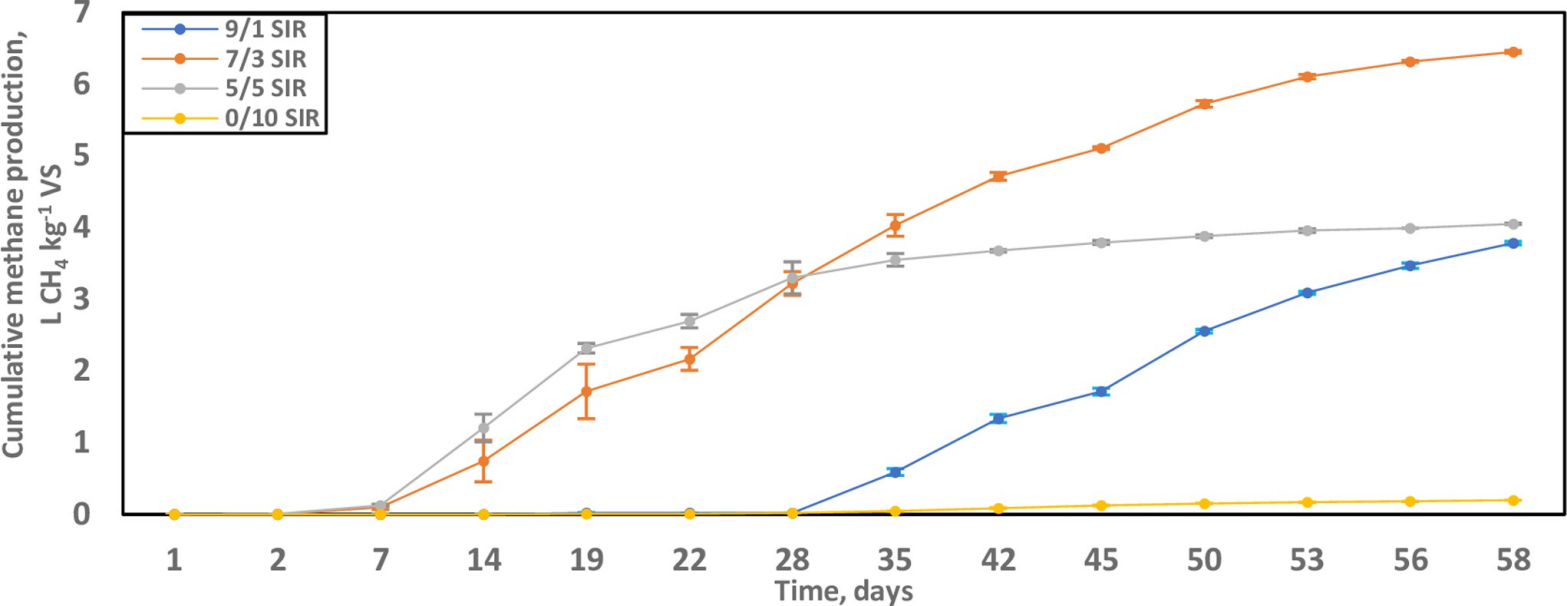
Cumulative methane production (L CH_4_ kg^-1^ VS) during the co-digestion of grape marc and cheese whey at 45°C over 58 days. The substrate-to-inoculum (S/I) ratios were 0/10 [yellow]; 5/5 [grey]; 7/3 [orange]; and 9/1 [blue]. Data are presented as averages with standard error.

The highest methane production was observed in reactors containing 7/3 S/I ratio, with cumulative methane production of 6.45 L CH_4_ kg^-1^ VS (5.94 NL CH_4_ kg^-1^ VS in STP) (Fig 2). Methane yields reduced to 4.05 L CH_4_ kg^-1^ VS (3.73 NL CH_4_ kg^-1^ VS in STP) in the reactor containing the 5/5 S/I ratio and was further reduced to 3.79 L CH_4_ kg^-1^ VS (3.49 NL CH_4_ kg^-1^ VS in STP) in 9/1 S/I reactors. When comparing reactors with the 7/3 and 5/5 S/I ratios, the cumulative methane yields were matched on day 28 beyond which 7/3 S/I ratios produced an additional 75% production over the remainder of the incubation (Fig 2). In terms of SMP values in reactors seeded with 5/5 and 9/1 S/I ratios, it can be concluded that the substrate at these S/I ratios values did not exert inhibitory or overloading effects on the inoculum [11]. Motte et al. [30] demonstrated that S/I ratio is a determining factor only during the start-up phase of digestion. As treatment proceeds, the SMP curve becomes a function of the total solids content. The slow start-up of reactors at 9/1 S/I ratio may also be attributed to the predominance of slowly digestible lignocellulosic biomass and the greater inhibitory effect of total ammoniacal nitrogen [2, 44, 45]. The Van Soest fractionation of wheat straw indicated cellulose, hemicellulose, and lignin concentrations of 38–44% TS, 30–36% TS, 6.5–6.9% TS, respectively [30]. Similarly high values were obtained by Ma et al. (2019) for cellulose (45% TS), hemicellulose (25% TS), and lignin (12% TS) in rape straw [12].

Endogenous methane production was minimal, considering the SMP curve on the digestions for 0/10 S/I ratio (0.20 L CH_4_ kg^-1^ VS when wet; 0.18 NL CH_4_ kg^-1^ VS in STP). Moreover, a large inoculum dose (based on wet weight) lowered the lag time to biomethanation in providing additional biocatalysts, higher initial total volatile acids, and a strong reaction buffer [2, 12]. Conversely, digestions were prone to significant lag, susceptible to feedstock overloading, and even digestion failure [2]. When assessing the three S/I levels of digestion, apart from the blank assays, digesters operated at 5/5 S/I ratio had sufficient inoculation to reduce the start-up time (Fig 2). However, excessive inoculum on those digesters utilised feedstock space, which quickly reached maximum production rate with a subsequently decreased VMPR profile [46]. In reactors with a 7/3 S/I ratio, there was inherent optimisation where a threshold inoculum mass inoculated a substrate. Further lowering of the inoculum dose to 9/1 S/I ratio may have resulted in the lowering of pH and accumulation of VFA’s, thus decreasing biomethanation [2].

Holliger et al. [11] recommended that the contribution of VS from the inoculum be greater than that from the substrate to minimise risks of reactor acidification or inhibitory effects [47]. For most applications, VS-inoculum was between two- and four-fold higher than the VS-substrate, corresponding to 1/2 and 1/4 S/I ratio, respectively. For readily degradable substrates such as food wastes where the accumulation of volatile acids could be inhibitory, S/I ratios lower than or equal to 1/4 are suggested. Finally, S/I ratios greater than or equal to 1/1 can be envisaged for similar substrates recalcitrant to biodegradation (Fig 2; [11]).

In the batch digestion of corn stover, Xu et al. [2] observed the highest cumulative methane yields of 239 and 200 L CH_4_ kg^-1^ VS at the S/I ratios of 2/1 and 4/1, respectively, for optimal performance profiles. Kafle et al. [48] established that a 2/1 S/I ratio was ideal for standard mesophilic and thermophilic temperature regimes in the treatment of Chinese cabbage; biogas yields were 677 mL gas kg^-1^ VS and 639 mL gas kg^-1^ VS for at 35.5°C and 55°C, respectively. Similarly, in the current study, the reactors that displayed the best SMP profiles (6.45 L CH_4_ kg^-1^ VS, highest value, when wet) were at 7/3 S/I ratio (alternatively written as 2.33/1 S/I ratio) in the treatment of the feedstock; this value is consistent with the reported optimal S/I ratio for lignocellulosic digestion.

### Volumetric methane productivity rate (VMPR)

**Fig 3:**
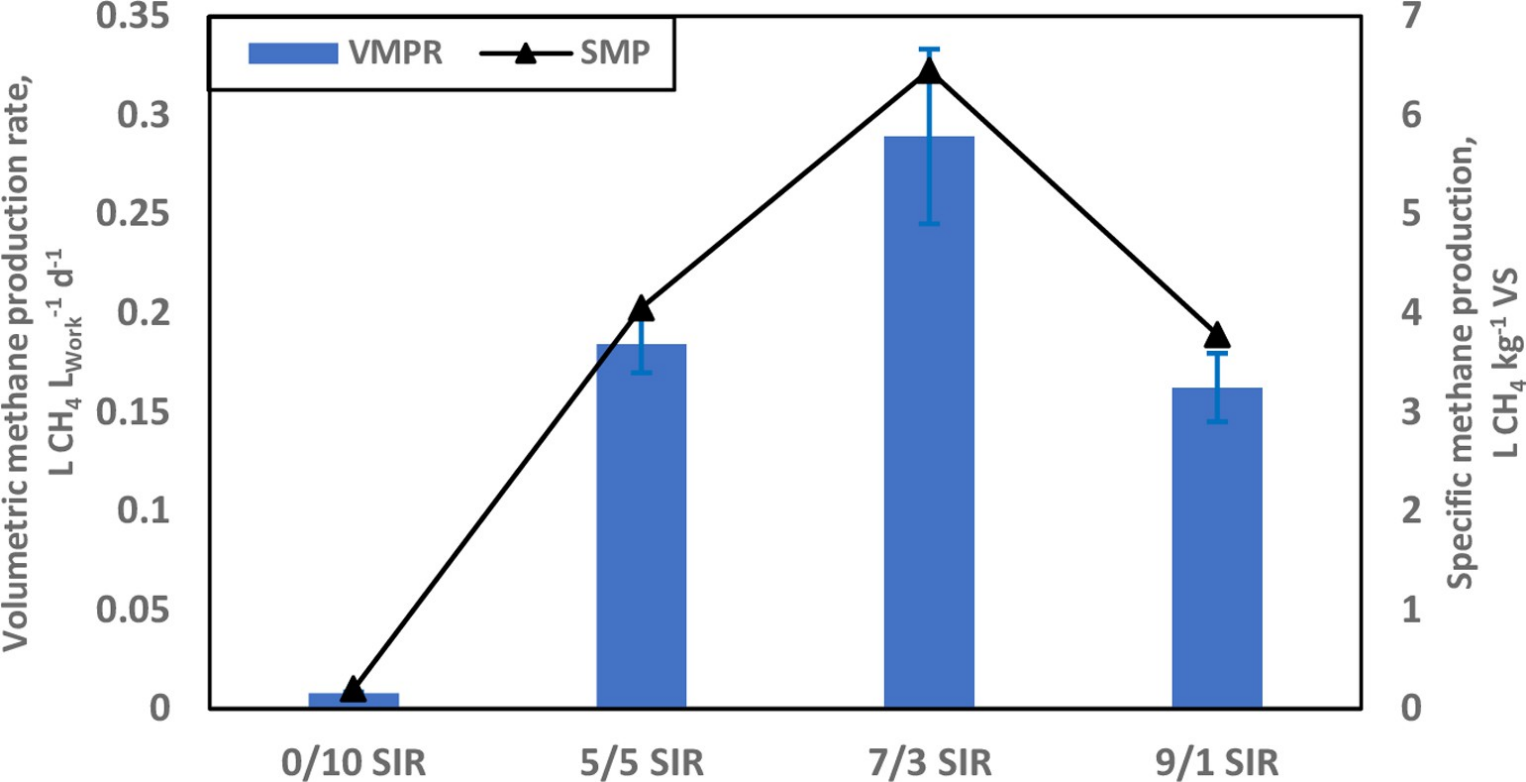
Experimental volumetric methane production rate (VMPR, L CH_4_ LWork-1 d^-1^) and the cumulative specific methane production (SMP, L CH_4_ kg^-1^ VS) from digestion setups with grape marc and cheese whey as mixed substrate (S) at different substrate-to-inoculum (S/I) ratios over 58 days of SS-AD. Data are presented as averages with standard error.

The VMPR of digesters at different S/I ratios is shown in Fig 3 The four levels of inoculum doses exhibited a normal distribution similar to the overall SMP curve, peaking at 7/3 S/I ratio. The highest observed VMPR was 0.289±0.044 L CH_4_ L_Work_^-1^ d^-1^ at 7/3 S/I ratio, significantly higher (p < 0.05) than those of 0.008±0.001, 0.184±0.015, and 0.175±0.017 L CH_4_ L_Work_^-1^ d^-1^ for digesters at S/I of 0/10, 5/5, and 9/1, respectively. Overall, optimal methane production were achieved in digesters at a 7/3 S/I ratio (Fig 3). The kinetics of the agri-industrial feedstock of this study was characterised by optimal digestion, inoculum consistency and stable operation revolving around a well-defined central S/I [47]. However, Ma et al. [12] did not observe a conclusive pattern of VMPR and SMP in the digestion of corn stover at variable S/I ratios; namely 2/3, 1/1, 2/1, 3/1, and 4/1, possibly because the inoculum fraction took up a larger working volume in reactors at low S/I, correspondingly decreasing the organic load for operation. The highest VMPR (0.42 L CH_4_ L_Work_^-1^ d^-1^) was observed for the digestion at 2/1 S/I ratio. Consequently, further optimisation of the S/I ratio was recommended to improve the SMP of the digestion of lignocellulosic feedstock, thus lowering the inoculum dosage at initially low S/I ratio and improve economics. Also, digestions with the smallest inoculum fraction (4/1 S/I ratio, wet weight basis) exhibited the smallest SMP and VMPR values [12]. This trend was confirmed in the present study of the co-digestion of GM and CW where the 9/1 S/I digestions produced the least SMP (3.79 L CH_4_ kg^-1^ VS) and VMPR (0.175 L CH_4_ L_Work_^-1^ d^-1^) values; whereas peak SMP (6.45 L CH_4_ kg^-1^ VS) and VMPR (0.289 L CH_4_ L_Work_^-1^ d^-1^) were reached at 7/3 S/I (Fig 3).

In contrast to the common industry practice of minimising inoculum dose (wet weight basis) for feedstock treatment, VMPR aims at an efficient reactor operation by optimising the bio-catalytic capacity that mediates the biomethanation of substrates. The identification of a critical limit of S/I ratio thus holds the potential for a substantial economical treatment configuration and operation [49].

### AD feedstock and digestate characterisation

#### Total solids (TS) and chemical oxygen demand (CODt)

The initial total solid concentration of the mixed feedstocks did not vary significantly (p > 0.05) and remained in the range of 20–30% TS across all reactor setups. However, the initial CODt (wet weight basis) ranged between 40 and 90 g L^-1^ (Table 2). The CODt removal efficiency ranged between 12–47% with the 7/3 S/I ratio exhibiting the lowest CODt removal. The lack of upstream particle screening before anaerobic treatment coupled with the absence of a filtering step before CODt determination resulted in a large initial concentration range (Table 2). Generally, pretreatment of the lignocellulosic biomass is routinely performed to accelerate hydrolysis and improve COD solubilisation [39]. Such pretreatment interventions can be thermal (low or high temperature, hydrothermal, and steam explosion), mechanical (sonication, and microwave irradiation), biological, chemical, or an assortment [50]. However, the benefits of minimising the manipulation of the feedstock for screening, filtering and pretreatment may potentially translate into further cost savings and improved commercial potential when scaling up treatment operations [10, 23]. The relatively stable organics level (12.73% COD removal, lowest) on the 7/3 S/I ratio can be linked to the inhibited rate of acidogenesis coupled with the generally slow methanogenesis in these digesters (Table 2; [51]). The lack of pretreatment on the GM-based feedstock may have allowed for the presence of large organic particle sizes conducive to slow hydrolysis, and a slow volatile fatty acids (VFAs) uptake, resulting in slow reactor acidification and concomitantly high biomethanation (Fig 3; [51, 52]). Kim et al. [52] established that at 35°C, COD removal is repressed due to the hydrolysates not readily converted to VFAs, indicating that the acidogenesis was rate-limiting. In addition, the slow process of secretion of exoenzymes involved in the solubilisation of organic polymers, sterically incompatible molecules or highly crystalline molecules coupled to transport across bacterial cytoplasmic membranes may delay fermentation [53]. Overall, the lack of pretreatment of GM and CW feedstocks resulted in slow hydrolytic and fermentative stages (low COD removal) upstream, in contrast to otherwise fast and inhibitory downstream AD biological processes, resulting in enhanced subsequent methanogenesis in digesters operated at 7/3 S/I ratio.

**Table 2:** Assessment of physicochemical characteristics after 58 days of treatments at 45°C. The mixed feedstocks were grape marc and cheese whey in ratio 3/1, respectively, before and after digestion at variable substrate-to-inoculum (S/I) ratio: 0/10, 5/5, 7/3, and 9/1. Values recorded as mean ± SE (n = 3).

Parameter	Unit	0/10 S/I		5/5 S/I		7/3 S/I		9/1 S/I	
		Bef.	Aft.	Bef.	Aft.	Bef.	Aft.	Bef.	Aft.
TS*	%	27.6 ±1.57	24.5 ±0.23	24 ±1.6	29.6 ±0.18	26.1 ±1.61	27.1 ±1.81	27.7 ±0.39	31.9 ±3.45
VS^#^	%	19.6 ±1.56	20.8 ±1.09	21.1 ±0.91	22 ± 0.4	21 ±1.1	21.7 ±1.01	22.3 ±0.47	26.6 ±1.23
CODt	g L^-1^	70.5 ±4.95	40 ±4.2	42.5 ±2.12	34 ±7.1	55 ±0.1	48 ±1.4	89 ±2.8	47 ±4.2
CODt removal	%	—	43.26	—	20	—	12.73	—	47.19
TKN	g L^-1^	16.1 ±0.49	13.6 ±1.39	8.03 ±1.59	3.83 ±1.41	11.8 ±1.65	4.86 ±0.51	20.7 ±1.73	13.2 ±0.29
pH	—	8.44 ±0.16	8.94 ±0.03	7.89 ±0.09	9.11 ±0.01	8.20 ±0.11	9.13 ±0.04	7.53 ±0.01	8.61 ±0.01
EC	mS cm^-1^	48.7 ±0.21	51.4 ±3.18	29.9 ±3.82	37 ±3.5	31.7 ±1.06	38.7 ±1.34	35.7 ±3.61	41.5 ±2.26
Salinity	%	12.5 ±0.07	21 ±0.2	13 ±1.4	15 ±0.1	11 ±0.1	15 ±0.2	13.5 ±3.54	14.5 ±0.71
COD/N	—	4.38	2.95	5.29	8.88	4.66	9.88	4.30	3.57

TS, total solids; VS, volatile solids; CODt, total COD; TKN, total Kjeldahl nitrogen; EC, electrical conductivity.

*,#: differences among means of the groups were not statistically significant.

#### Nutrition

The C/N ratio is the standard indicator for substrate nutritional quality; low C/N values impede microbial metabolism and growth [54]. Traditionally, a digestion C/N ratio of 20–30 is regarded as optimal range for stable performance and high methane production for organic substrates [54–56]. However, the wide range of favourable C/N ratios across organic waste types, and the low correlation between C/N ratios and methane yields suggest that a much stronger explanatory variable than C/N ratio needs consideration in further analyses [57]. For example, enhanced SMP was achieved when the C/N ratios were 15–39 for corn stover ([5, 58]); 19–30 for wheat straw ([5, 59]); 17–35 for tree trimmings [60]; and 50–65 for maple and pine woods [5]. In addition, not all the carbon present in the substrate feedstock is completely available for biodegradation during AD (assumed in the C/N ratio) [61]. Instead, the measured effluent COD (relative to the initial concentration) represents a more accurate picture of the digestibility of the substrate [38, 62]; also, a combination of biotic factors and operating conditions such as particle size, temperature and S/I ratio all play a role in the conversion of extractable organic carbon to methane and carbon dioxide [30].

Biological treatment through AD increases process stability and enhances the COD/N ratio [62]. Consequently, a comparison of post-treatment COD/N trends captures the overall balance of carbon availability and buffering capacity (nitrogen content) for bacterial growth and ultimate biogas production [38, 62]. The final COD/N value observed was the highest (9.88) in the optimal 7/3 S/I setups and lowest (2.95) in the 0/10 S/I blank assays. The digestate COD/N values positively correlated with both VMPR and SMP curves, indicating that the specific nutritional contents of digesters had a bearing on reactor performance in this specific co-digestion study of unamended grape marc and cheese whey (Fig 3). Previously, Da Ros et al. [62] established a biological process range for a COD/N ratio of 17.5–20 for winery waste digestate (post-treatment) in the anaerobic co-digestion of winery wastewater sludge and wine lees at 37°C and 55°C treatment temperatures.

#### pH

pH, initially in the range of 7.53–8.44, increased during the digestion, irrespective of reactor setups; the final pH reached 9.13, the highest, in 7/3 S/I digesters (Table 2). During fermentation, the growing partial pressure of CO_2_ in the headspace volume can combine with the oxygen trapped in the aqueous phase to produce bicarbonate ions [63]. This chemical behaviour increases the pH of the digestate, thus maintaining suitable conditions for prolonged and stable methane production without requirements for pH adjustment (Table 2). Shi et al. [64] established that the alkalinity resulting from the combined physicochemical composition of the lignocellulosic biomass and that of the inoculum may positively impact the stabilisation of the operating pH during high-solid anaerobic treatment. In contrast, various reports documented a suitable pH range of 6.5–7.5 for enhanced biogas production [65–67]. Whilst this trend is common, pH represents the sum of all the biochemical reactions occurring in a particular medium. However, Nolla-Ardèvol et al. [68] established that continuous biogas production of high methane purity (96% CH_4_) is possible from the digestion of microalga species at 35°C in extreme haloalkaline conditions (pH 10, 2.0 M Na^+^). As pointed out previously, the complexity of waste co-digestion and inoculum size, type and source can balance an overall stable pH specific to the reactor performance under consideration [2, 64, 69].

#### Electrical conductivity (EC) and salinity

Initial EC ranged between 40 and 50 mS cm^-1^, increasing to significantly higher final values (p < 0.05). The 7/3 and 5/5 S/I digesters registered an average 22% increase in EC (Table 2). Robles et al. [70] observed a linear relationship between the bicarbonate ions, SMP, and EC throughout anaerobic treatment [71]. The increased pH range of effluents on all reactor setups suggest the formation of bicarbonates (Table 2). There are significant economic and environmental considerations in attaining higher conductivity for sustainable methane production without addition of exogenous and polluting conductive materials such as graphene and magnetite [72–75].

The initial salinity variations were not statistically significant across S/I groups; however, the final salt concentrations were higher than the initial values. The optimal 7/3 S/I showed the lowest initial salinity at 11%. An initially low salinity may be beneficial, potentially stimulating methane production over the baseline control (Table 2). For example, the salinity of 15 g L^-1^ contributed to a cumulative SMP greater than the reference value during anaerobic treatment of macroalgae [76]; salt concentrations as high as 85 g L^-1^ severely inhibited methanogenesis. Therefore, high salinity levels owing to mineral salts such as light metals (calcium, sodium, magnesium and potassium) exert bacteriostatic, in some cases bactericidal effects, on microorganisms due to increased osmotic pressure, detrimental to cellular integrity [76, 77]. Zhao et al. [78] demonstrated that an adequate initial salinity can solubilise the digestate, releasing organics from previously bound and granular states. This biochemical feedback loop is understood to release additional mineral salts to the medium, increasing the final salinity and SMP [78]. Moreover, high salinity without prior acclimation may disrupt inoculum enzyme functions, ultimately leading to reactor failure. As methane yield is inversely linked to salinity, an adequate initial salinity as well as microbial tolerance is essential for stable biomethanation [76].

### Elemental analyses

The results of elemental characterisation are reported in Table 3. Grape marc contains a substantial fraction of carbon, predominant in the substrate co-digestion ratio 3/1 GM/CW (w/w). Inconsistencies between replicates (> 0.3% SE, standard error; 95% CI, confidence interval) were mostly attributable to heterogeneous samples. Where duplicates were consistent, the samples were homogeneous. Consequently, in digesters where substrate was evaluated, the elemental carbon removal efficiencies were 2.03%, 15.07%, and 13.84% in digesters at 5/5, 7/3, and 9/1 S/I ratio, respectively, after treatment. The organic carbon removal efficiency for biomethanation was understandably low because of the slowly degradable lignocellulosic portion that generally requires extended digestion times for reaction completion; further, an estimated 5–10% of inlet carbon was diverted from methane to microbial metabolism [79, 80]. The carbon removal (15.07%) was the highest in the 7/3 S/I digestions, corresponding to an optimal biomass conversion to methane.

**Table 3:** Elemental characteristics (CHNOS) of the substrates and digestate at varying substrate-to-inoculum (S/I) ratio through the course of treatment.

	Unmixed feedstock			Before treatment				After treatment			
	GM	CW	Inoculum (0/10 S/I)		5/5 S/I	7/3 S/I	9/1 S/I	0/10 S/I	5/5 S/I	7/3 S/I	9/1 S/I
Elemental											
composition^a^											
%N	2.19 ±1.07	1.67 ±0.18	2.34 ±0.01		2.69 ±1.02	2.83 ±1.43	2.97 ±1.84	2.54 ±0.59	2.16 ±0.19	3.19 ±0.75	2.33 ±1.22
%C	47.3 ±2.98	31.1 ±2.21	39.5 ±0.13		49.2 ±8.07	53.1 ±11.3	57.1 ±14.5	45.5 ±1.12	48.2 ±1.13	45.1 ±0.09	49.2 ±0.78
%H	5.78 ±0.34	4.82 ±0.55	5.24 ±0.26		6.33 ±1.01	6.77 ±1.42	7.20 ±1.82	5.81 ±0.04	6.17 ±0.31	5.73 ±0.16	6.49 ±0.25
%S	0.03 ±0.00	0.39 ±0.08	0.23 ±0.04		0.17 ±0.01	0.15 ±0.01	0.12 ±0.01	0.16 ±0.05	0.10 ±0.05	0.11 ±0.07	0.10 ±0.07
%O^b^	44.7 ±2.25	62.1 ±2.50	52.7 ±0.34		41.6 ±6.05	37.2 ±14.2	32.6 ±18.8	46.0 ±0.51	43.4 ±1.30	45.9 ±1.08	41.9 ±0.26
C, H, N, O											
coefficients^c^											
a	25.2	21.73	19.69		21.34	21.89	22.43	20.9	26.03	16.49	24.64
b	36.95	40.41	31.35		32.94	33.49	33.94	32.02	40	25.15	39
c	17.86	34.49	19.74		13.54	11.49	9.61	15.85	17.57	12.58	15.73
d^d^	1	1	1		1	1	1	1	1	1	1
Stoichiometry	C_25_H_37_O_18_N	C_22_H_40_O_35_N	C_20_H_31_O_20_N		C_21_H_33_O_14_N	C_22_H_34_O_12_N	C_22_H_34_O_10_N	C_21_H_32_O_16_N	C_26_H_40_O_18_N	C_17_H_25_O_13_N	C_25_H_39_O_16_N
Theoretical											
yields											
(m^3^ kg^-1^ VS)											
B_th_	0.8838	0.5618	0.7387		0.9205	0.9932	1.0681	0.8511	0.9011	0.8433	0.9199
M_th_	0.4342	0.1789	0.3172		0.4757	0.5392	0.6036	0.4119	0.4586	0.4024	0.4812

^**a**^ values are expressed as grams of element per 100 grams of sample;

^**b**^ the oxygen content was obtained as complement to 100 and purified from the water fraction;

^**c**^ the sulphur content was not considered because it is assumed negligible;

^**d**^ nitrogen was the element with the minimum number of moles; B_th_ is the theoretical biogas yield; M_th_ is the theoretical methane yield.

Individual elemental composition of unmixed feedstock with the corresponding maximum theoretical yields are also shown. N (nitrogen), C (carbon), H (hydrogen), and S (sulphur) were determined on dry weight basis. Values recorded as mean ± standard error.

The theoretical methane production potential showed that CW (0.1789 m^3^ CH_4_ kg^-1^ VS) mono-digestion was lower than that of GM digested alone (0.4342 m^3^ CH_4_ kg^-1^ VS) based on elemental characterisation, thus confirming the positive impact of feedstock co-digestion on reactor performance (Table 3; [81]). The calculated theoretical biogas yields before and after digestions at various S/I ratios, based on the remaining available organic substrates for reaction, were not significantly different (p < 0.05).

The mixing of CW and GM feedstock in digesters at the various S/I ratios significantly improved methane yields, in excess of values achieved of the feedstocks taken individually (Table 3). Various reports have confirmed the positive contribution of co-digestion on digester performance [82, 83]. In a parallel study of the co-digestion of GM and CW, Kassongo et al. [81] confirmed that a digester’s stable performance can be further enhanced through adequate operational controls such as system architecture, inoculum source, increased digestion temperature (45°C), higher treatment capacity and long incubation period (120 days), among others; the calculated substrate utilisation efficiency reached > 60%, based on CH_4_ extraction alone from organics without consideration for transformation of the biodegradable matrix into CO_2_. We postulate, therefore, that the determined optimal 7/3 S/I for the co-digestion of GM and CW can potentially reach higher carbon removal values if scaled up to similar large-scale digester operations [81].

### Kinetic simulations

**Fig 4:**
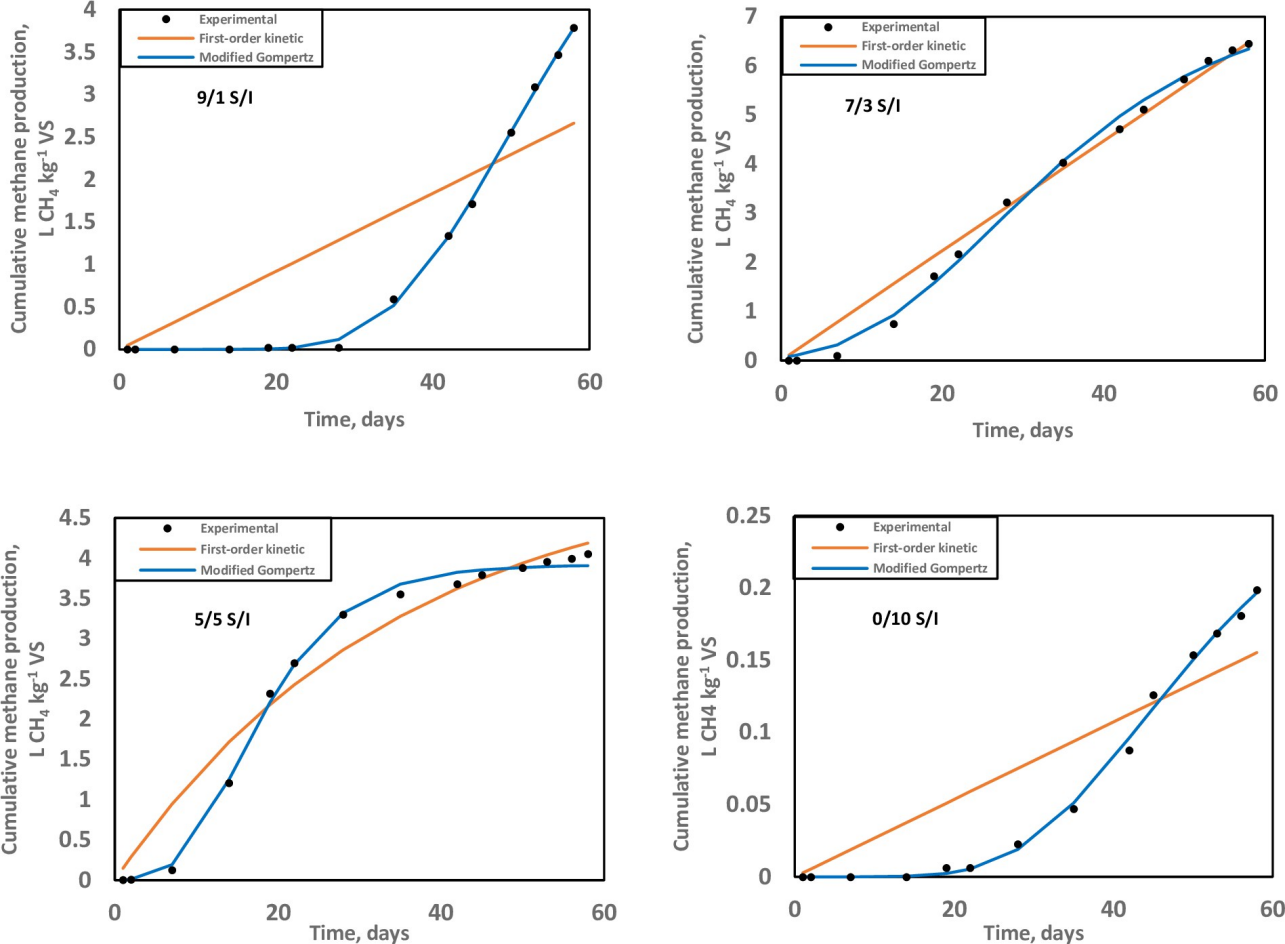
Simulations of the cumulative methane production, L CH_4_ kg^-1^ VS, using the first-order regression model (orange); and the modified Gompertz model (blue).

To characterise the kinetic degradation behaviour, two model structures for SMP simulation, the first-order kinetic and the modified Gompertz, were fitted to the experimental data. Parameter optimisation is obtained by minimising the SSD (sum of squared deviations) between measured and corresponding simulated values-based. There were generally unreasonable first-order kinetic model parameters of overestimated *B*_*0*_, *k*, and weak test statistic SSD across setups, irrespective of S/I. However, the modified Gompertz model showed better agreement with the data. The variations between the data and simulation were minimal, 0.06–3.57% across reactor setups, when fitted with the modified Gompertz model (Table 4). Interestingly, the differences between the data and simulation were 0.36% and 3.36% in the 9/1 and 5/5 S/I digesters, respectively, for the first-order kinetic model. In contrast, the modified Gompertz model showed larger variations with the data whilst the test statistic SSD was minimised in the particular reactor setups (Table 4). Moreover, the root mean squared error (RMSE), the square root of the average squared difference between the experimental and predicted values by the model, corresponded to SSD trends; the lower the RMSE, the better the model. There was close data approximation for digesters at 9/1 and 5/5 S/I ratios by both the first-order kinetic and the modified Gompertz models (Fig 4). The modified Gompertz model parameterisation also displayed a lag time of 7–34 days (Table 4) across reactor setups.

**Table 4:** Parameters and goodness fit obtained with the evaluated models, first-order kinetic and the modified Gompertz, for the waste treatment over 58 days at 45°C.

Simulation	Unit	9/1 S/I	7/3 S/I	5/5 S/I	0/10 S/I
***First-order kinetic model***					
B_0_	L CH_4_ kg^-1^ VS	1627	1556	5.147	80.02
k	d^-1^	2.82E-05	7.18E-05	0.028	3.34E-05
Sum of squared deviations (SSD)	—	7.912	1.542	1.449	0.016
Root mean squared error (RMSE)	L CH_4_ kg^-1^ VS	0.751	0.331	0.321	0.033
Measured methane yield	L CH_4_ kg^-1^ VS	3.784	6.451	4.052	0.198
–day 58					
Predicted methane yield	L CH_4_ kg^-1^ VS	2.663	6.474	4.188	0.155
–day 58					
Difference between measured and predicted methane yield	%	29.62	0.361	3.362	21.90
(in absolute value)					
***Modified Gompertz model***					
B_0_	L CH_4_ kg^-1^ VS	6.631	7.397	3.917	0.293
λ	d	34.03	9.446	7.730	28.12
R_m_	L CH_4_ kg^-1^ VS d^-1^	0.160	0.160	0.198	0.006
Sum of squared deviations (SSD)	—	0.021	0.334	0.094	0.000
Root mean squared error (RMSE)	L CH_4_ kg^-1^ VS	0.039	0.154	0.082	0.004
Measured methane yield	L CH_4_ kg^-1^ VS	3.784	6.451	4.052	0.198
–day 58					
Predicted methane yield	L CH_4_ kg^-1^ VS	3.782	6.342	3.907	0.196
–day 58					
Difference between measured and predicted methane yield	%	0.061	1.688	3.577	1.061
(in absolute value)					

The substrate-to-inoculum (S/I) ratios were 9/1, 7/3, 5/5, and 0/10.

The feedstock digested for biomethanation consisted of grape marc known for the presence of phenolic compounds and alcohol that are slowly amenable to degradation, hence the intervening lag phase during treatment [43, 84]. At both ends of the S/I spectrum (viz. 0/10 and 9/1), there was a noticeable lag time. An increase in the inoculum fraction reduced the lag and improved reactor performance (Fig 4). Similarly, Koch et al [31] established that too little inoculum concentration in relative proportion to substrate introduced a lag time on the SMP curves; higher inoculum digestions led to better-fitting of common anaerobic digestion models. Additionally, a previous study by Kafle et al. [85] suggested increasing the S/I as a suitable mechanism to lower the lag time and increase biogas production.

## Conclusions

This study demonstrated that grape marc, in a co-digestion with cheese whey, is a suitable feedstock for methane production and concomitant waste treatment without requirements for pretreatment, alkalinity control, or mixing during reactor operation. Overall, biochemical data identified similarities in the digestate profiles of 5/5 and 7/3 S/I ratios. However, the optimal S/I ratio was at 7/3 (wet weight), coinciding with peak 6.45 L CH_4_ kg^-1^ VS, significantly greater than the peak methane production of 4.05 L CH_4_ kg^-1^ VS in digesters at 5/5 S/I ratio. This cumulative methane production in digesters at 7/3 S/I positively correlated with a volumetric methane production rate of 0.289±0.044 L CH_4_ L_Work_^-1^ d^-1^. In digesters at S/I levels lower than optimal, nutrient was limiting, whereas feedstock overloading was a possible factor when reduced inoculum dosage at S/I levels greater than optimal. Further digestate analyses revealed that the final pH, electrical conductivity, and salinity levels were all increased at the termination of treatment, irrespective of the S/I ratio. The 7/3 S/I digesters showed the lowest influent salinity; however, the highest effluent pH, and COD/N ratio. The modified Gompertz model validated the experimental data with a parameterisation of 9.4 days for lag time to steady-state methane production. Furthermore, this study embodied the practical workings of a commercial-scale digester where incoming mixed feedstock can conveniently be co-digested on wet weight basis, with reduced reliance on a more complex TS indicator.

The streamlined preliminary trial characterised by a minimal energy input only for the digestion temperature regime along with the optimal S/I can potentially translate into significant economic value when unencumbered operations maximise methane recovery from feedstock per cubic volume of digesters. Future work will explore the impact of bacterial community engineering at the optimal substrate-to-inoculum ratio on digester performance when increasing the feedstock treatment capacity.

## Supporting information

S1 File (PDF)
