# Supplementary material for: Substrate-to-inoculum ratio drives solid-state anaerobic digestion of unamended grape marc and cheese whey
Source: PLoS One. 2022 Jan 27;17(1):e0262940. doi: 10.1371/journal.pone.0262940 (PMC8794148; doi:10.1371/journal.pone.0262940)
Supplement: S1 File — (PDF) [file pone.0262940.s001.pdf]

Elemental Microanalysis Report

Sample ID: 1
Date Received: 16 October 2020
Date Analysed: 21 October 2020

Analysis Results:

Sample ID	Weight (mg)	N%	C%	H%	S%
1	3.601	1.44	49.44	6.017	0.03
	2.107	2.95	45.22	5.541	0.03
Theoritical values	-	-	-	-	-

Sample ID: 2
Date Received: 16 October 2020
Date Analysed: 21 October 2020

Analysis Results:

Sample ID	Weight (mg)	N%	C%	H%	S%
2	4.637	1.80	29.51	4.426	0.442
	5.426	1.54	32.63	5.204	0.335
Theoritical values	-	-	-	-	-

Sample ID: 3
Date Received: 16 October 2020
Date Analysed: 21 October 2020

Analysis Results:

Sample ID	Weight (mg)	N%	C%	H%	S%
3	1.792	2.34	39.40	5.063	0.254
	3.643	2.33	39.59	5.424	0.198
Theoritical values	-	-	-	-	-

Sample ID: 4
Date Received: 16 October 2020
Date Analysed: 21 October 2020

Analysis Results:

Sample ID	Weight (mg)	N%	C%	H%	S%
4	3.793	2.96	44.66	5.781	0.195
	2.785	2.12	46.24	5.836	0.123
Theoritical values	-	-	-	-	-

Sample ID: 5
Date Received: 16 October 2020
Date Analysed: 21 October 2020

Analysis Results:

Sample ID	Weight (mg)	N%	C%	H%	S%
5	4.186	2.02	48.96	6.387	0.139
	2.152	2.30	47.36	5.947	0.062
Theoritical values	-	-	-	-	-

Sample ID: 6
Date Received: 16 October 2020
Date Analysed: 21 October 2020

Analysis Results:

Sample ID	Weight (mg)	N%	C%	H%	S%
6	2.445	3.72	45.18	5.839	0.163
	2.009	2.66	45.04	5.612	0.058
Theoritical values	-	-	-	-	-

Sample ID: 7
Date Received: 16 October 2020
Date Analysed: 21 October 2020

Analysis Results:

Sample ID	Weight (mg)	N%	C%	H%	S%
7	4.347	1.47	49.70	6.667	0.053
	4.267	3.19	48.60	6.32	0.147
Theoritical values	-	-	-	-	-

Comments:

- Sample was stored in a fridge before analysis.
- Values are expressed as grams of element per 100 grams of sample. For organic standard materials the trueness 95% confidence limit of the technique is ± 0.3% with a precision of ± 0.2%. Please note that the presence of water or other solvents, filter paper fibres or other foreign substances in the sample will result in appreciable deviations from the expected theoretical values.
